# Case Report: Acute inferior myocardial infarction and third-degree atrioventricular block in a patient with hyperthyroidism

**DOI:** 10.3389/fcvm.2025.1692856

**Published:** 2025-11-12

**Authors:** Qing Qiu, Fen Li, RuYi Zhong, Miao Li

**Affiliations:** Department of Endocrinology and Metabolic Diseases, Liuyang People’s Hospital, Liuyang, Hunan, China

**Keywords:** hyperthyroidism, acute inferior myocardial infarction, third-degree AV block, coronary angiography, abnormal thyroid function, coronary artery disease

## Abstract

We describe the management of a middle-aged male with hyperthyroidism who presented acute inferior myocardial infarction (AIMI) complicated by third-degree atrioventricular (AV) block. The symptoms of AIMI in the patient were atypical. However, his atypical presentation raised clinical suspicion. The electrocardiogram (ECG) was immediately conducted to confirm the patient's AIMI and third-degree AV block. Further coronary angiography revealed that the patient's right coronary artery was completely occluded at the second bend. A stent was placed in the right coronary artery. Following anti-thyroid therapy, the patient's clinical symptoms improved. This case underscores the importance of recognizing atypical presentations in hyperthyroid patients and is followed by a literature review exploring the mechanisms by which thyroid dysfunction influences coronary artery disease (CAD).

## Introduction

The incidence of hyperthyroidism or acute myocardial infarction (AMI) individually is relatively high; however, their concurrent presentation is relatively rare (1, 2). Hyperthyroidism is one of the endocrine diseases, and its occurrence can cause changes in multiple organs. Hyperthyroidism can cause or aggravate cardiovascular diseases, including arrhythmia, CAD and heart failure, thereby increasing morbidity and mortality (3). Thyroid hormones play a significant role in increasing heart rate and enhancing myocardial contractility. However, patients with hyperthyroidism may experience aggravated symptoms due to increased myocardial oxygen demand and/or coronary artery spasm, thereby triggering hemodynamic changes and cardiovascular events. AMI refers to a condition where, due to the persistent and severe state of myocardial ischemia, some myocardium undergoes acute necrosis. The research on the mutual influence mechanism between hyperthyroidism and AMI is worthy of our further exploration.

## Case report

A 48-year-old male was admitted to the hospital due to heat intolerance, excessive sweating, palpitations, hand tremors and weight loss for over 4 months. He developed chest tightness 2 days prior to admission. He reported that he had been diagnosed with hyperthyroidism during a physical examination before October, but he did not pursue any treatment. More than 4 months ago, the patient had hyperthyroidism with FT3 of 20.86 pmol/L (reference range 3.85–6.3), FT4 of 39.35 pmol/L (12.8–21.3) and TSH <0.001 mU/L (0.75–5.6). A history of treatment with propylthiouracil for 1 month. As he was feeling well, he then stopped taking the medicine. The symptoms recurred and were accompanied by chest tightness for 2 days, prompting him to seek medical care. At the time of presentation, he denied having chest pain or shortness of breath.

The patient has a history of gouty arthritis. There was no family history of cardiovascular disease and thyroid disease, and no personal history of chronic diseases such as hypertension and diabetes. Psychosocially, the patient is a self-employed individual with a secondary education level, who does not smoke or drink alcohol. He lives with his wife and children, and reports a harmonious family relationship. The patient complained of recent general discomfort accompanied by poor sleep. He denied significant work-related or life stressors. The onset of these symptoms is suspected to be related to hyperthyroidism. While the patient has a good family support system, he tends to delay seeking medical help and has a history of poor adherence to medical advice. He presented to the hospital only after an acute worsening of his symptoms.

Physical examination: Blood pressure: 120/64 mmHg, heart rate: 65 beats per minute, the thyroid gland was not enlarged or tender, had a medium texture, and no vascular bruits were auscultated. No significant abnormalities were detected on cardiopulmonary auscultation. The patient's relatively slow heart rate was inconsistent with his thyrotoxic symptoms.

**Figure 1:**
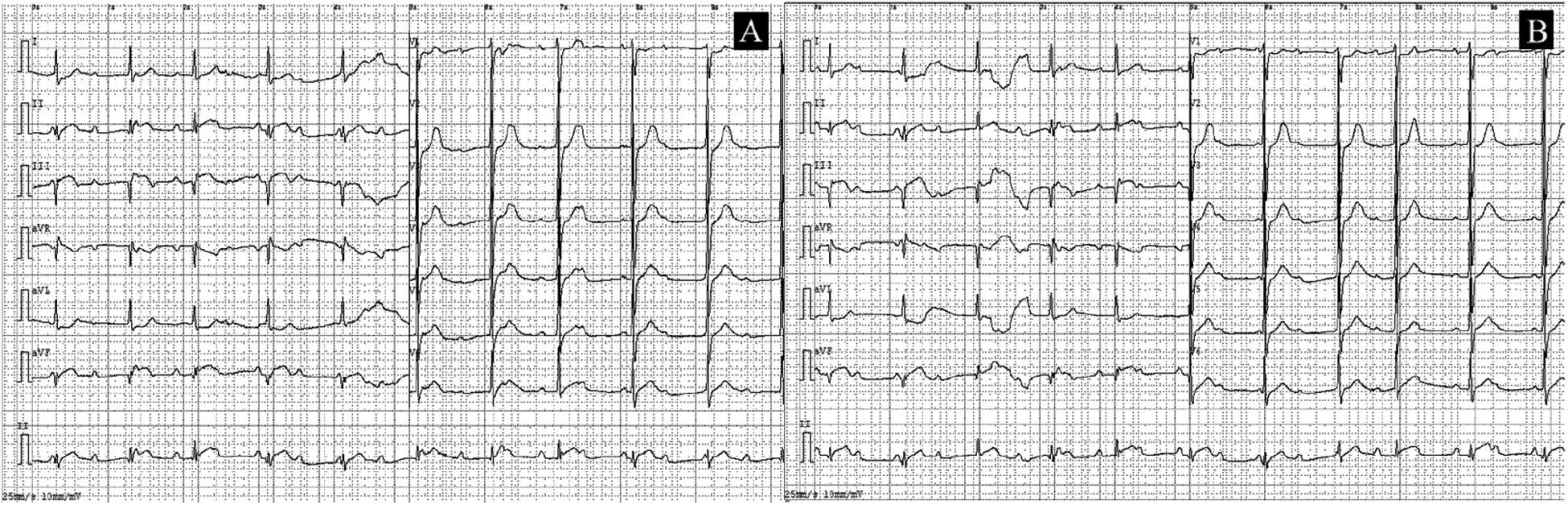
ECGs obtained at the time of presentation. **(A)** The initial ECG shows sinus rhythm with pathological Q waves and ST-T changes in leads II, III, and aVF; third-degree AV block could not be excluded. **(B)** A follow-up ECG obtained 15 min later.

An immediate ECG showed sinus rhythm with pathological Q waves and ST-segment elevation in leads II, III, and aVF, along with third-degree AV block (Figure 1). Serum cardiac biomarkers were significantly elevated, with troponin T at 1,197 pg/mL (0–14), CK at 98 U/L (50–310), CK-MB at 18 U/L (0–20), myoglobin at 32.5 μg/L (0–70), pro-BNP II 4,307 pg/mL (0–125) and D-dimer levels 0.69 mg/L (0–0.55). Thyroid function tests were obtained and revealed: FT3 9.17 pg/mL (2–4.4), FT4 3.97 ng/mL (0.93–1.7), TSH <0.0005 uIU/mL (0.27–4.2), TPOAB 508 IU/mL (<34), TGAB 255.1 IU/mL (<115), TRAB 6.92 IU/L (<1.75). Glucose 5.84 mmol/L, and plasma lipids showed surprisingly low concentrations (total cholesterol 2.59 mmol/L, triglycerides 0.86 mmol/L, LDL-cholesterol 1.59 mmol/L and HDL cholesterol 0.5 mmol/L). White blood cell count (WBC) 10.88 × 109/L (3.5–9.5), Neutrophil count (NEUT) 7.94 × 109/L (1.8–6.3), C-reactive protein (CRP) 96.19 mg/L (0–6), Interleukin-6 (IL-6) 87.78 pg/mL (<7), renal and hepatic function were within normal limits.

**Figure 2:**
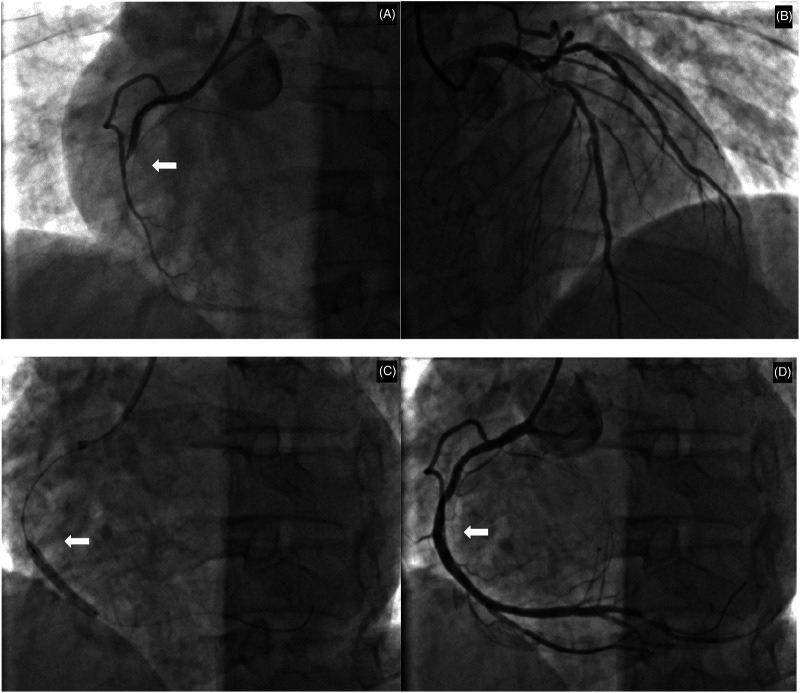
Coronary angiographic findings and interventional procedure. **(A)** Initial angiography revealing total occlusion at the second bend of the right coronary artery. **(B)** Angiography of the left coronary artery showing the proximal left anterior descending and circumflex arteries. **(C)** Balloon angioplasty being performed at the site of the occlusion. **(D)** Final result following successful stent deployment, with restored blood flow and resolution of the stenosis. White arrows indicate the primary lesion or intervention site.

The patient's hyperthyroidism was uncontrolled due to self-discontinuation of medication. Consequently, there is an elevated risk of emergency percutaneous coronary intervention (PCI), coupled with the potential for thyroid storm. He subsequently provided informed consent and underwent the procedure. Coronary angiography findings (Figure 2): Right coronary artery: Diffuse stenosis is evident in the proximal and mid-segments, with the most severe degree reaching 60%. Complete occlusion is observed at the second bend, and distal antegrade blood flow is TIMI grade 0. Other blood vessels show atherosclerotic plaque stenosis lesions. Accordingly, a stent was implanted in the right coronary artery. Comprehensive echocardiographic assessment demonstrated: (LVEDV: 52 mm, LAS: 38 mm, RVD: 33 mm, IVSD: 10 mm, EF: 64%) Left atrial enlargement and asynchronous ventricular wall motion with mild hypokinesis of the inferior wall, accompanied by impaired left ventricular diastolic function. Thyroid ultrasound findings indicated diffuse parenchymal abnormalities and multiple nodules in the left lobe of the thyroid gland, classified as TI-RADS category 3. Postoperatively, the patient received treatment for coronary artery dilation and antiplatelet therapy including aspirin 100 mg once daily and clopidogrel 75 mg once daily. Lipid-lowering therapy included atorvastatin 20 mg combined with ezetimibe 10 mg nightly. Additional cardioprotective medications included nicorandil, sacubitril/valsartan, metoprolol succinate sustained-release tablets, and Shexiang Baoxin Pills (a Chinese herbal medicine for CAD). For hyperthyroidism management, methimazole 10 mg once daily was administered, along with multivitamin supplements and selenium yeast tablets. The patient's chest tightness resolved significantly post-procedure.

**Figure 3:**
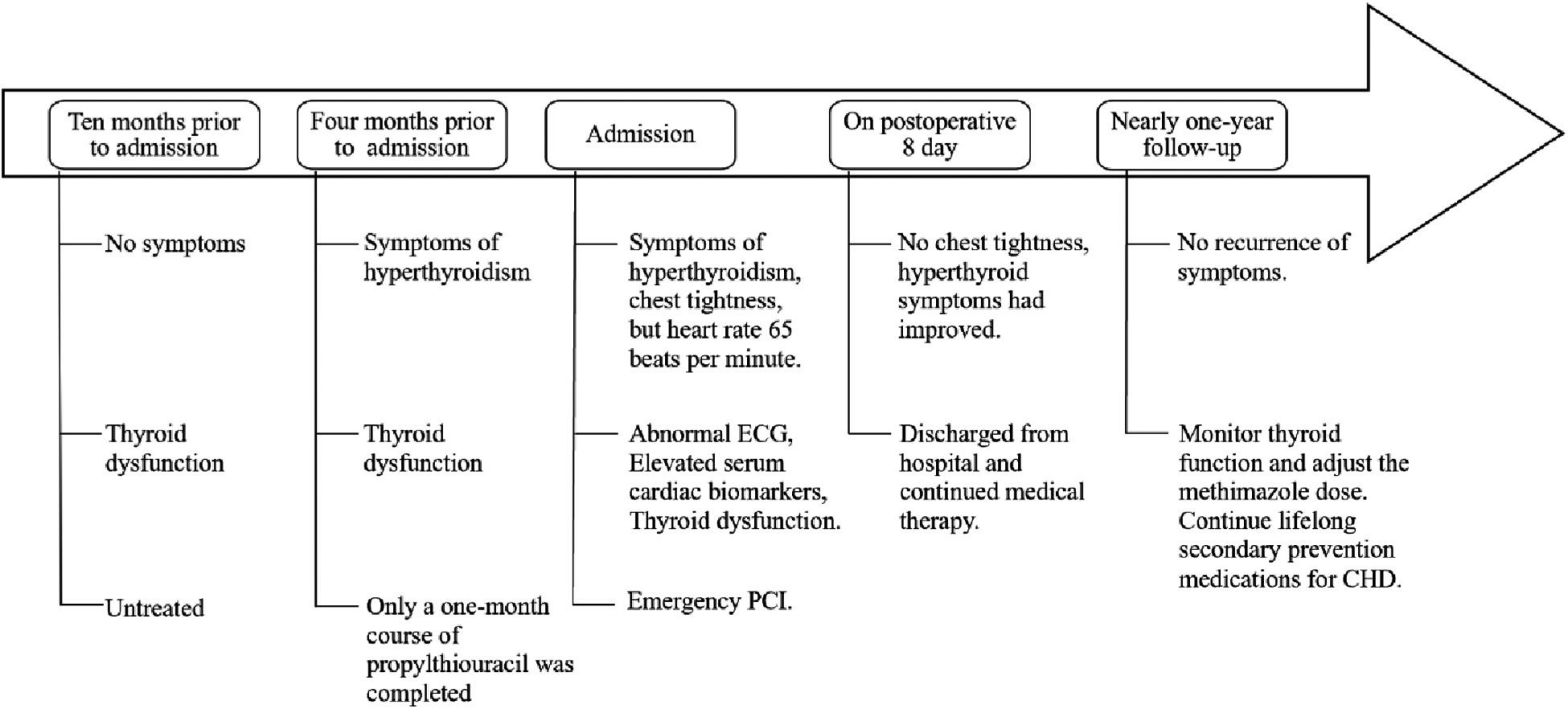
Timeline.

After discharge, the patient continued regular outpatient follow-up at our hospital for thyroid function monitoring (Table 1), with methimazole doses adjusted accordingly. He remained asymptomatic throughout the subsequent follow-up period, with no recurrence of chest discomfort. The patient acknowledged that this experience underscored the critical importance of seeking timely medical care and adhering to treatment recommendations. He reflected that his initial non-adherence to hyperthyroidism treatment likely contributed to his subsequent AIMI, and the uncontrolled thyroid condition significantly heightened the peri-procedural risk, provoking substantial anxiety for both him and his family. Nevertheless, he proceeded with the PCI based on trust in the clinical team, which resulted in a successful outcome. Consequently, the patient committed to closer adherence to medical advice, including regular thyroid function monitoring and prompt medication adjustments. He specifically pledged to avoid arbitrarily discontinuing his therapy. The patient's timeline is shown in Figure 3.

**Table 1:** After the operation, patients are advised to attend regular follow-up appointments at our hospital's outpatient department for reevaluation of the thyroid function tests and related antibody levels, as well as for appropriate adjustment of the methimazole dosage.

Date/Project	TSH (uIU/mL)	FT3 (pg/mL)	FT4 (ng/mL)	TRAB (IU/L)	TPOAB (IU/mL)	TGAB (IU/mL)	Methimazole dosage/daily (mg)
2024.07.28	<0.0005	9.17	3.97	6.92	508	255.1	10
2024.09.05	<0.005	7.22	1.73	10.98	587		20
2024.10.03	0.00629	3.27	0.55				10
2024.11.01	1.17	3.23	0.661				5
2024.12.02	0.233	4.51	1.14	10.98			10
2024.12.31	0.0192	3.88	1.08				10
2025.02.17	2.5	2.9	0.682	5.54	477		5
2025.06.06	1.88	3.26	0.887	3.07	308		5

FT3, free triiodo-thyronine; FT4, free thyroxine; TSH, thyroid-stimulating Hormone; TPOAB, thyroid peroxidase antibody; TGAB, thyroglobulin antibody; TRAB, thyroid-stimulating hormone receptor antibody.

## Discussion

This patient is a middle-aged male with a history of gouty arthritis. He has no prior medical history of hypertension, diabetes, hyperlipidemia, cardiovascular disease, or other related comorbidities, and he is a non-smoker and non-drinker. Recently, he presented with symptoms indicative of a hypermetabolic state. Thyroid function tests revealed significantly elevated levels of FT3 and FT4, along with suppressed TSH, confirming the diagnosis of hyperthyroidism. Over the past 2 days, the patient reported experiencing chest tightness and discomfort. However, there was no associated chest pain, dyspnea, sense of impending doom, or radiating pain. These atypical symptoms could easily be mistaken for cardiovascular manifestations secondary to hyperthyroidism rather than AMI. During physical examination, the patient's heart rate was found to be within normal limits but relatively bradycardic for his hypermetabolic state. A bedside ECG was promptly performed, revealing ST-segment elevation in leads II, III and aVF, along with third-degree AV block. These findings raised suspicion for an AIMI, which could potentially explain the patient's uncharacteristically slow heart rate despite hyperthyroidism. Additionally, cardiac biomarkers of myocardial necrosis were markedly elevated. Further diagnostic evaluation via coronary angiography demonstrated complete occlusion at the second bend of the right coronary artery, with TIMI grade 0 distal antegrade flow. Echocardiographic assessment showed dyssynchronous ventricular wall motion and mildly reduced contractility in the inferior wall. Based on these findings, the patient was diagnosed with AIMI and third-degree AV block, and was concurrently diagnosed with hyperthyroidism. No significant stenosis or dilation of the occluded vessel was observed following administration of nitroglycerin prior to stent implantation. Given the presence of severe coronary atherosclerotic plaques and multivessel stenotic lesions, atherosclerosis-related AMI was determined as the underlying cause rather than coronary vasospasm, and percutaneous coronary intervention with stent implantation was carried out accordingly. While hyperthyroidism alone is rarely associated with AMI in the absence of fixed coronary disease or vasospasm, the co-occurrence of AMI and hyperthyroidism (particularly thyrotoxic storm) constitutes a high-risk clinical scenario. Although manageable with early diagnosis, this combination carries a significant risk of mortality if not promptly recognized and treated. To our knowledge, this represents one of the extremely rare cases of hyperthyroidism accompanied by concurrent AMI and third-degree AV block. This unique clinical scenario presentation provides valuable insights for the clinical diagnosis and management of such complex cases.

Whether hyperthyroidism combined with AMI is primarily attributable to hyperthyroidism or involves other contributing factors warrants in-depth clinical consideration. Thyroid hormones play a crucial role in maintaining cardiovascular homeostasis. However, the precise mechanisms by which they influence the cardiovascular system remain incompletely understood. Clinically, both thyroid hormones excess and deficiency can induce or exacerbate cardiovascular conditions such as arrhythmia, atherosclerosis, dyslipidemia, and heart failure, thereby increasing the risk of early onset and mortality associated with cardiovascular disease (4). The reported incidence of hyperthyroidism coexisting with angina pectoris due to coronary heart disease ranges from 0.5% to 20%, whereas its coexistence with AMI is relatively rare (1, 2).

The pathogenesis of CAD in patients with hyperthyroidism may be attributed to several mechanisms:

First, Coronary Artery Thrombosis: Persistent coronary artery spasm and vascular endothelial injury can elevate thromboxane A2 (TXA2) levels, promoting platelet activation and aggregation, which may ultimately lead to thrombus formation. Additionally, during hyperthyroidism, increased cell membrane permeability and red blood cell deformability, along with elevated plasma factor VIII levels, contribute to a hypercoagulable state (5). Patients with hyperthyroidism often develop atrial fibrillation, which predisposes them to left atrial mural thrombi, embolization, and subsequent coronary artery embolism (6, 7).

Second, Increased Myocardial Oxygen Demand: Elevated thyroid hormone levels enhance sympathetic nervous system activity, increase catecholamine secretion, and potentiate vasoconstrictive effects of adrenaline and noradrenaline (8). Concurrently, hypermetabolism and elevated catecholamines reduce atrial natriuretic peptide levels, diminishing its vasodilatory capacity (9) and predisposing the coronary arteries to spasm. Such vasospasm, coupled with thyroid hormone-induced increases in myocardial contractility and heart rate, can critically elevate myocardial oxygen demand. Direct stimulation of the myocardium by thyroid hormones exacerbates this effect, potentially leading to myocardial ischemia and AMI. International case reports have documented coronary artery spasm in hyperthyroid patients (10, 11). Accurate differentiation between hyperthyroidism-induced coronary vasospasm and atherosclerosis-induced AMI is crucial, as their management strategies differ substantially. Reversible stenosis observed after nitroglycerin administration during coronary angiography serves as direct evidence of coronary artery spasm. For patients presenting with typical chest pain but without significant angiographic stenosis, intravascular ultrasound (IVUS) or optical coherence tomography (OCT) may be employed prior to revascularization to differentiate between atherosclerosis and vasospastic disease (12). In this case, no significant stenosis or dilation of the occluded vessel was observed following administration of nitroglycerin prior to stent implantation. This suggests that the infarction was likely due to underlying severe atherosclerosis, which may have been exacerbated by the hyperthyroid state. Most case reports (13, 14) attribute angina pectoris or AMI to coronary artery spasm from hyperthyroidism, whereas this case highlights that acute coronary syndrome in these patients can also be caused by severe atherosclerosis (15).

Third, Hyperglycemia and Insulin Resistance: Hyperthyroidism accelerates intestinal glucose absorption via T4-induced phosphorylation and inhibits glycogen synthesis, leading to elevated blood glucose levels. Studies indicate the presence of insulin resistance in these patients (16), increasing their susceptibility to impaired glucose tolerance or diabetes mellitus both of which are recognized risk equivalents for CAD. However, in this particular case, the patient exhibited normal glucose metabolism upon admission, ruling out this mechanism as a primary contributor to AMI. Additionally, the patient exhibited elevated WBC and NEUT, along with increased CRP and IL-6 levels indicative of systemic inflammation. This inflammatory state likely exacerbated vascular endothelial injury, contributing to the development of AMI. Further clinical investigation is warranted to confirm this hypothesis.

In clinical settings, when coronary angiography is required for patients with uncontrolled thyroid function, iodinated contrast media (ICM) are typically used. However, the administration of iodine-based contrast media may trigger or worsen thyroid storm (TS) in hyperthyroid individuals. Furthermore, there have been documented cases indicating the occurrence of thyroid storm following the administration of ICM during cardiac angiography in patients with AMI who had not undergone prior thyroid function assessment (17). Based on the 2021 European Thyroid Association guidelines on managing thyroid dysfunction induced by ICM (18) and the Chinese Expert Consensus on the Clinical Diagnosis and Treatment of Cardiovascular Diseases Complicated with Thyroid Dysfunction (19), the following recommendations are proposed: Elective procedures should be considered for patients with active, uncontrolled thyroid dysfunction and significant clinical manifestations. In emergency situations requiring urgent coronary angiography, non-iodinated contrast agents may be considered to minimize complications. Furthermore, one study has shown (20) that diluting iodinated contrast 1:1 with normal saline and limiting the total volume to 15 ml resulted in no complications when administered via femoral access to facilitate emergency interventions.

In this case, the patient received standard secondary prevention therapy for CAD alongside active management of hyperthyroidism. Regular follow-up of thyroid function and dose adjustments of methimazole contributed to improved clinical outcomes and disease recovery. This case underscores the rarity and clinical significance of third-degree AV block secondary to AMI in a hyperthyroid patient. It highlights the necessity of routine thyroid function screening in AMI patients and emphasizes the critical importance of a “comprehensive management” strategy that combines acute revascularization with concurrent endocrine intervention.

## Conclusion

Clinically, when patients with hyperthyroidism present with cardiovascular symptoms, initial suspicion often falls on thyrotoxic cardiomyopathy rather than CAD, potentially leading to diagnostic delays and missed opportunities for timely intervention. Therefore, a comprehensive evaluation comprising medical history, physical examination, biochemical tests, ECG, and dynamic monitoring of myocardial necrosis markers is essential. When clinically indicated, coronary angiography should be performed for definitive diagnosis. In life-threatening emergencies such as AMI, the use of iodine-free or low-dose iodine contrast agents may be appropriate, followed by vigorous hydration to facilitate iodine excretion. After thorough explanation of the risks, informed consent must be obtained. For the management of hyperthyroidism complicated by CAD, antithyroid medications should be administered concurrently with standard cardiac therapies to reduce basal metabolic rate. This dual approach effectively lowers myocardial oxygen demand and addresses the underlying pathophysiology, thereby improving patient outcomes.

## Data Availability

The original contributions presented in the study are included in the article/Supplementary Material, further inquiries can be directed to the corresponding author.
