# Supplementary figures and images for: Case Report: Acute inferior myocardial infarction and third-degree atrioventricular block in a patient with hyperthyroidism
Source: Front Cardiovasc Med. 2025 Nov 12;12:1692856. doi: 10.3389/fcvm.2025.1692856 (PMC12647106; doi:10.3389/fcvm.2025.1692856)

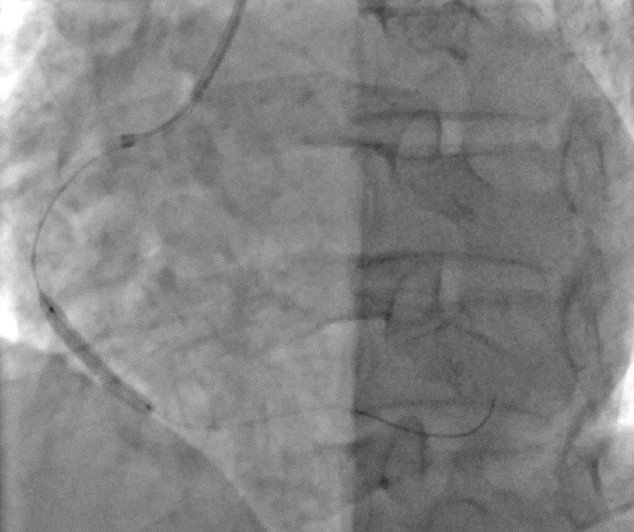

Supplement: Supplementary file 1 [file Image1.jpeg]

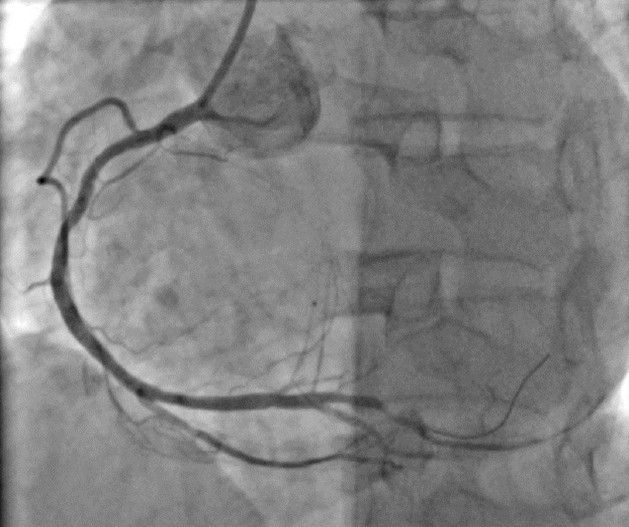

Supplement: Supplementary file 2 [file Image2.jpeg]
